# Bis[μ-1,2-bis­(1*H*-imidazol-1-ylmeth­yl)benzene-κ^2^ *N* ^3^:*N* ^3′^]disilver(I) 3-carboxyl­ato-4-hydroxy­benzene­sulfonate methanol solvate trihydrate

**DOI:** 10.1107/S1600536809030268

**Published:** 2009-08-08

**Authors:** Hong-Mei Sun, Yun-Chao Chi, Hai-Yan Liu

**Affiliations:** a Department of Chemistry and Pharmaceutical Engineering, Suihua University, Suihua 152061, People’s Republic of China

## Abstract

In the title compound, [Ag_2_(C_14_H_14_N_4_)_2_](C_7_H_4_O_6_S)·CH_3_OH·3H_2_O, the complex dication has a binuclear structure in which each Ag^I^ ion is two-coordinated in a slightly distorted linear coordination geometry. The two Ag^I^ atoms are bridged by two 1,2-bis­[(1*H*-imidazol-1-yl)meth­yl]benzene (IBI) ligands, forming a 22-membered ring. In the dication, π–π inter­actions are observed between the imidazole rings with centroid–centroid distances of 3.472 (3) and 3.636 (3) Å. In the crystal, the uncoordinated water mol­ecules, anions and methanol solvent mol­ecules are linked into chains along the *b* axis by O—H⋯O hydrogen bonds. In addition, π–π inter­actions are observed between the benzene rings of the IBI ligands, with a centroid–centroid distance of 3.776 (2) Å. The sulfonate group is disordered over two orientations with occupancies of 0.676 (12) and 0.324 (12).

## Related literature

For the design and synthesis of silver(I) sulfonates, see: Cote & Shimizu (2003 ▶); Ma *et al.* (2005 ▶). For silver(I) sulfonate compounds modified by secondary nitro­gen-based ligands, see: Cote & Shimizu (2004 ▶); Liu *et al.* (2007 ▶). For Ag—N bond distances in N-containing Ag^I^ compounds, see: Li *et al.* (2006 ▶).
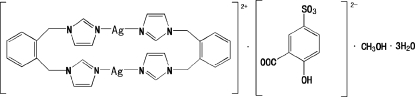


## Experimental

### 

#### Crystal data

[Ag_2_(C_14_H_14_N_4_)_2_](C_7_H_4_O_6_S)·CH_4_O·3H_2_O
*M* *_r_* = 994.58
Triclinic, 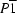

*a* = 8.9959 (18) Å
*b* = 13.947 (3) Å
*c* = 16.008 (3) Å
α = 102.71 (3)°
β = 92.37 (3)°
γ = 91.34 (3)°
*V* = 1956.5 (7) Å^3^
*Z* = 2
Mo *K*α radiation
μ = 1.12 mm^−1^
*T* = 293 K
0.18 × 0.15 × 0.12 mm

#### Data collection

Rigaku R-AXIS RAPID diffractometer
Absorption correction: multi-scan (*ABSCOR*; Higashi, 1995 ▶) *T*_min_ = 0.771, *T*_max_ = 0.869
19149 measured reflections
8696 independent reflections
5684 reflections with *I* > 2σ(*I*)
*R*_int_ = 0.030

#### Refinement

*R*[*F* ^2^ > 2σ(*F* ^2^)] = 0.043
*wR*(*F* ^2^) = 0.113
*S* = 1.04
8696 reflections
569 parameters
15 restraints
H atoms treated by a mixture of independent and constrained refinement
Δρ_max_ = 0.48 e Å^−3^
Δρ_min_ = −0.41 e Å^−3^

### 

Data collection: *PROCESS-AUTO* (Rigaku, 1998 ▶); cell refinement: *PROCESS-AUTO*; data reduction: *PROCESS-AUTO*; program(s) used to solve structure: *SHELXS97* (Sheldrick, 2008 ▶); program(s) used to refine structure: *SHELXL97* (Sheldrick, 2008 ▶); molecular graphics: *SHELXTL-Plus* (Sheldrick, 2008 ▶); software used to prepare material for publication: *SHELXL97*.

## Supplementary Material

Crystal structure: contains datablocks global, I. DOI: 10.1107/S1600536809030268/ci2841sup1.cif

Structure factors: contains datablocks I. DOI: 10.1107/S1600536809030268/ci2841Isup2.hkl

Additional supplementary materials:  crystallographic information; 3D view; checkCIF report

## Figures and Tables

**Table:** 

Ag1—N5	2.105 (3)
Ag1—N3	2.121 (3)
Ag2—N4	2.089 (3)
Ag2—N7	2.091 (3)

**Table:** 

N5—Ag1—N3	176.76 (13)
N4—Ag2—N7	172.60 (13)

**Table 2:** Hydrogen-bond geometry (Å, °)

*D*—H⋯*A*	*D*—H	H⋯*A*	*D*⋯*A*	*D*—H⋯*A*
O1*W*—H1*B*⋯O3*W*	0.85 (6)	2.35 (5)	2.892 (9)	122 (4)
O2*W*—H2*A*⋯O1	0.86 (5)	2.52 (6)	3.063 (7)	122 (6)
O3—H4*A*⋯O1	0.86 (4)	1.71 (3)	2.486 (6)	149 (5)
O7—H7*A*⋯O6^i^	0.84 (1)	1.88 (2)	2.697 (6)	164 (4)
O2*W*—H2*B*⋯O1*W*^ii^	0.86 (5)	2.24 (5)	2.790 (9)	122 (4)
O3*W*—H3*A*⋯O3*W*^iii^	0.85 (6)	2.52 (4)	3.053 (10)	122 (2)

Symmetry codes: (i) 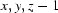; (ii) 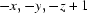; (iii) 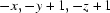.

**Fig. 1.**
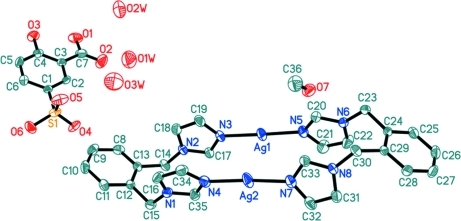
The aymmetric unit of the title compound, showing the atomic numbering scheme. Displacement ellipsoids are drawn at the 30% probability level. Only the major disorder component of the C7H4O6S2- ion is shown.

**Fig. 2.**
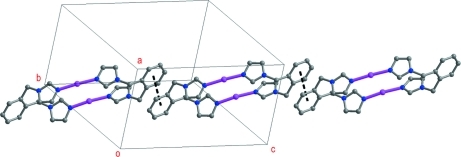
View of the formation of a chain through π-π interactions in the title compound. The anions, methanol and water molecules have been omitted for clarity.

## supplementary crystallographic information

### Comment

The design and synthesis of silver(I) sulfonates have attracted intense interests of chemists (Cote & Shimizu, 2003; Ma *et al.*, 2005). Some silver(I) sulfonate compounds, modified by secondary nitrogen-based ligands, have been reported (Cote & Shimizu, 2004; Liu *et al.*, 2007). In most of these silver(I) sulfonate complexes, the sulfonate ligand acts as a counter-anion. Herein, we present a new silver-sulfonate complex (I), namely, [Ag_2_(IBI)_2_]*L*^.^CH_3_OH^.^3H_2_O, where IBI is 1,2-bis[(1*H*-imidazol-1-yl)methyl]benzene and *L* is 3-carboxy-4-hydroxybenzenesulfonic acid.

Selected bond distances and angles are listed in Table 1. The contents of the asymmetric unit is shown in Fig.1. The silver complex shows a binuclear structure, where each of Ag^I^ atom has a slightly distorted linear geometry and is coordinated by two N atoms from the IBI ligands. The Ag—N bond distances are within the normal range and are comparable to those observed in N-containing Ag^I^compounds (Li *et al.*, 2006). Notably, the *L* ligand does not coordinate to the Ag^I^ center and acts as a counter-anion. In the complex dication, π–π interactions are observed between the imidazole rings with Cg1···Cg2 and Cg3···Cg4 distances of 3.472 (3) and 3.636 (3) Å, respectively; Cg1, Cg2, Cg3 and Cg4 are centroids of the N1/C16/C24/N4/C35, N2/C17/N3/C19/C18, N5/C20/N6/C22/C21 and N7/C32/C31/N8/C33, rings, respectively.

In the crystal, the lattice water molecules, sulfonate oxygen atoms, and hydroxyl oxygen atoms are involved in the formation of O—H···O hydrogen-bonded chains along the *b* axis (Table 2). In addition, π-π interactions between the benzene rings of the IBI ligands, with a Cg5···Cg6^iii^ distance of 3.776 (2) Å [Cg5 and Cg6 are centroids of the C8-C13 and C24-C29 rings, respectively; symmetry code: (iii) x, y, 1+z] are observed (Fig.2).

### Experimental

An aqueous solution (10 ml) of 3-carboxy-4-hydroxybenzenesulfonate anion (1 mmol) was added to solid Ag_2_CO_3_ (0.5 mmol) and stirred for several minutes until no further CO_2_ was given off. 1-(3-(1*H*-Imidazol-1-yl)methyl)benzyl)-1*H*-imidazole (1 mmol) was then added and a precipitate was formed. The precipitate was dissolved by ammonium hydroxide. Single crystals of the title compound were obtained by slow evaporation of the solution for 5 d at room temperature.

### Refinement

Hydroxy H atoms were located in a difference map and refined with a O—H distance restraint of 0.85 (1) Å. Water H atoms were located in a difference Fourier map and refined with O—H and H···H distance restraints of 0.85 (1) Å and 1.35 (1) Å, respectively, and with *U*_iso_(H) = 1.5*U*_eq_(O). At this stage, short H···H contacts involving water H atoms were observed but attempts to locate alternate positions for these H atoms failed. Hence, the H1A···H3B, H1B···H3B, H1A···H2B^ii^ and H3A···H3A^iii^ [symmetry code: (ii) -*x*,-*y*,1 - *z*); (iii): -*x*,1 - *y*,1 - *z*] distances were restrained to 2.20 (1) Å to avoid short H···H contacts. H atoms bonded to C atoms were positioned geometrically (C-H = 0.93 or 0.97 Å) and refined as riding, with *U*_iso_(H) = 1.2*U*_eq_(C). The sulfonate O atoms are disordered over two orientations with occupancies of 0.676 (12) and 0.324 (12).

### Crystal data

**Table:**

[Ag_2_(C_14_H_14_N_4_)_2_](C_7_H_4_O_6_S)·CH_4_O·3H_2_O	*Z* = 2
*M**_r_* = 994.58	*F*(000) = 1008
Triclinic, *P*1	*D*_x_ = 1.688 Mg m^−^^3^
Hall symbol: -P 1	Mo *K*α radiation, λ = 0.71073 Å
*a* = 8.9959 (18) Å	Cell parameters from 8696 reflections
*b* = 13.947 (3) Å	θ = 3.0–27.5°
*c* = 16.008 (3) Å	µ = 1.12 mm^−^^1^
α = 102.71 (3)°	*T* = 293 K
β = 92.37 (3)°	Block, colourless
γ = 91.34 (3)°	0.18 × 0.15 × 0.12 mm
*V* = 1956.5 (7) Å^3^	

### Data collection

**Table:**

Rigaku R-AXIS RAPID diffractometer	8696 independent reflections
Radiation source: fine-focus sealed tube	5684 reflections with *I* > 2σ(*I*)
graphite	*R*_int_ = 0.030
Detector resolution: 10.0 pixels mm^-1^	θ_max_ = 27.5°, θ_min_ = 3.0°
ω scans	*h* = −11→10
Absorption correction: multi-scan (*ABSCOR*; Higashi, 1995)	*k* = −18→18
*T*_min_ = 0.771, *T*_max_ = 0.869	*l* = −20→20
19149 measured reflections	

### Refinement

**Table:**

Refinement on *F*^2^	Primary atom site location: structure-invariant direct methods
Least-squares matrix: full	Secondary atom site location: difference Fourier map
*R*[*F*^2^ > 2σ(*F*^2^)] = 0.043	Hydrogen site location: inferred from neighbouring sites
*wR*(*F*^2^) = 0.113	H atoms treated by a mixture of independent and constrained refinement
*S* = 1.04	*w* = 1/[σ^2^(*F*_o_^2^) + (0.0508*P*)^2^ + 0.9392*P*] where *P* = (*F*_o_^2^ + 2*F*_c_^2^)/3
8696 reflections	(Δ/σ)_max_ = 0.001
569 parameters	Δρ_max_ = 0.48 e Å^−^^3^
15 restraints	Δρ_min_ = −0.41 e Å^−^^3^

### Special details

**Table:**

Geometry. All s.u.'s (except the s.u. in the dihedral angle between two l.s. planes) are estimated using the full covariance matrix. The cell s.u.'s are taken into account individually in the estimation of s.u.'s in distances, angles and torsion angles; correlations between s.u.'s in cell parameters are only used when they are defined by crystal symmetry. An approximate (isotropic) treatment of cell s.u.'s is used for estimating s.u.'s involving l.s. planes.
Refinement. Refinement of *F*^2^ against ALL reflections. The weighted *R*-factor *wR* and goodness of fit *S* are based on *F*^2^, conventional *R*-factors *R* are based on *F*, with *F* set to zero for negative *F*^2^. The threshold expression of *F*^2^ > 2σ(*F*^2^) is used only for calculating *R*-factors(gt) *etc*. and is not relevant to the choice of reflections for refinement. *R*-factors based on *F*^2^ are statistically about twice as large as those based on *F*, and *R*- factors based on ALL data will be even larger.

### Fractional atomic coordinates and isotropic or equivalent isotropic displacement parameters (Å^2^)

**Table:**

	*x*	*y*	*z*	*U*_iso_*/*U*_eq_	Occ. (<1)
Ag1	0.51631 (4)	0.61818 (2)	0.156865 (18)	0.05298 (11)	
Ag2	0.50633 (5)	0.87099 (3)	0.205113 (19)	0.06856 (14)	
C1	0.0311 (4)	0.3785 (3)	0.8229 (2)	0.0382 (8)	
C2	0.0830 (4)	0.2920 (3)	0.7783 (2)	0.0405 (8)	
H2	0.1572	0.2934	0.7398	0.049*	
C3	0.0281 (4)	0.2017 (3)	0.7887 (2)	0.0422 (8)	
C4	−0.0854 (4)	0.2010 (3)	0.8456 (3)	0.0480 (9)	
C5	−0.1369 (4)	0.2880 (3)	0.8914 (3)	0.0532 (10)	
H5	−0.2112	0.2869	0.9299	0.064*	
C6	−0.0797 (4)	0.3770 (3)	0.8810 (3)	0.0488 (9)	
H6	−0.1148	0.4356	0.9125	0.059*	
C7	0.0878 (5)	0.1075 (3)	0.7394 (3)	0.0658 (12)	
C8	0.3217 (4)	0.6528 (3)	0.6160 (2)	0.0485 (10)	
H8	0.3240	0.5845	0.6048	0.058*	
C9	0.2240 (4)	0.7004 (4)	0.6736 (3)	0.0563 (12)	
H9	0.1607	0.6645	0.7006	0.068*	
C10	0.2201 (5)	0.8008 (4)	0.6910 (2)	0.0585 (11)	
H10	0.1543	0.8334	0.7300	0.070*	
C11	0.3149 (4)	0.8536 (3)	0.6501 (2)	0.0474 (9)	
H11	0.3124	0.9218	0.6618	0.057*	
C12	0.4132 (4)	0.8056 (3)	0.5921 (2)	0.0357 (7)	
C13	0.4166 (4)	0.7038 (3)	0.5745 (2)	0.0364 (7)	
C14	0.5186 (4)	0.6475 (3)	0.5104 (2)	0.0495 (9)	
H14A	0.6148	0.6818	0.5165	0.059*	
H14B	0.5334	0.5830	0.5224	0.059*	
C15	0.5127 (4)	0.8669 (3)	0.5488 (2)	0.0492 (10)	
H15A	0.5279	0.9321	0.5854	0.059*	
H15B	0.6090	0.8371	0.5413	0.059*	
C16	0.3078 (4)	0.8984 (3)	0.4464 (3)	0.0550 (11)	
H35	0.2310	0.9104	0.4841	0.066*	
C17	0.5375 (4)	0.6265 (3)	0.3517 (2)	0.0437 (8)	
H17	0.6408	0.6244	0.3517	0.052*	
C18	0.3123 (4)	0.6337 (4)	0.3954 (3)	0.0584 (11)	
H18	0.2307	0.6372	0.4296	0.070*	
C19	0.3091 (5)	0.6254 (4)	0.3102 (3)	0.0620 (12)	
H19	0.2234	0.6233	0.2752	0.074*	
C20	0.4847 (4)	0.6288 (3)	−0.0366 (2)	0.0393 (8)	
H20	0.3817	0.6331	−0.0367	0.047*	
C21	0.7098 (5)	0.6150 (4)	0.0021 (3)	0.0675 (13)	
H21	0.7945	0.6082	0.0354	0.081*	
C22	0.7108 (4)	0.6219 (4)	−0.0795 (3)	0.0626 (13)	
H22	0.7932	0.6204	−0.1129	0.075*	
C23	0.5088 (4)	0.6425 (3)	−0.1888 (2)	0.0374 (8)	
H23A	0.4109	0.6703	−0.1836	0.045*	
H23B	0.4987	0.5782	−0.2274	0.045*	
C24	0.6103 (3)	0.7078 (2)	−0.22626 (19)	0.0323 (7)	
C25	0.7119 (4)	0.6636 (3)	−0.2848 (2)	0.0470 (9)	
H25	0.7166	0.5955	−0.2990	0.056*	
C26	0.8063 (4)	0.7204 (4)	−0.3223 (2)	0.0577 (12)	
H26	0.8736	0.6902	−0.3616	0.069*	
C27	0.8006 (5)	0.8198 (4)	−0.3016 (3)	0.0605 (12)	
H27	0.8650	0.8577	−0.3261	0.073*	
C28	0.7001 (4)	0.8649 (3)	−0.2446 (2)	0.0496 (10)	
H28	0.6956	0.9331	−0.2318	0.059*	
C29	0.6049 (4)	0.8095 (2)	−0.2058 (2)	0.0346 (7)	
C30	0.4982 (4)	0.8632 (3)	−0.1433 (2)	0.0419 (8)	
H30A	0.4784	0.9265	−0.1566	0.050*	
H30B	0.4048	0.8257	−0.1494	0.050*	
C31	0.6986 (4)	0.9128 (3)	−0.0229 (3)	0.0568 (11)	
H31	0.7754	0.9309	−0.0540	0.068*	
C32	0.7026 (5)	0.9151 (3)	0.0608 (3)	0.0576 (11)	
H32	0.7846	0.9357	0.0983	0.069*	
C33	0.4839 (4)	0.8623 (3)	0.0121 (2)	0.0454 (9)	
H33	0.3854	0.8395	0.0085	0.055*	
C34	0.3000 (5)	0.9002 (3)	0.3616 (3)	0.0586 (11)	
H36	0.2161	0.9142	0.3311	0.070*	
C35	0.5229 (5)	0.8640 (3)	0.3931 (2)	0.0496 (9)	
H324	0.6225	0.8480	0.3888	0.060*	
C36	0.0543 (6)	0.8125 (4)	−0.0864 (3)	0.0770 (14)	
H36A	0.0016	0.8051	−0.0372	0.115*	
H36B	−0.0107	0.7942	−0.1368	0.115*	
H36C	0.0879	0.8798	−0.0793	0.115*	
N1	0.4492 (3)	0.8757 (2)	0.46511 (19)	0.0432 (7)	
N2	0.4585 (3)	0.6358 (2)	0.42210 (18)	0.0410 (7)	
N3	0.4505 (4)	0.6207 (2)	0.28299 (18)	0.0448 (7)	
N4	0.4359 (4)	0.8780 (2)	0.3290 (2)	0.0524 (8)	
N5	0.5701 (4)	0.6192 (2)	0.03033 (19)	0.0451 (7)	
N6	0.5658 (3)	0.6314 (2)	−0.10393 (17)	0.0341 (6)	
N7	0.5693 (4)	0.8829 (2)	0.0834 (2)	0.0511 (8)	
N8	0.5583 (3)	0.8786 (2)	−0.05409 (18)	0.0384 (7)	
O1	0.0306 (4)	0.0287 (2)	0.7521 (3)	0.0817 (11)	
O2	0.1844 (5)	0.1116 (3)	0.6878 (3)	0.1196 (18)	
O3	−0.1455 (4)	0.1150 (3)	0.8567 (2)	0.0720 (9)	
H4A	−0.094 (5)	0.068 (3)	0.829 (3)	0.09 (2)*	
O7	0.1786 (3)	0.7512 (2)	−0.0952 (2)	0.0607 (8)	
H7A	0.147 (4)	0.6923 (12)	−0.110 (2)	0.045 (12)*	
O1W	0.1305 (6)	0.2310 (5)	0.5406 (4)	0.1396 (19)	
H1A	0.0362 (19)	0.234 (3)	0.533 (6)	0.209*	
H1B	0.156 (7)	0.285 (2)	0.575 (5)	0.209*	
O2W	0.0029 (6)	−0.0585 (5)	0.5588 (3)	0.1278 (17)	
H2A	−0.058 (6)	−0.040 (7)	0.599 (3)	0.192*	
H2B	−0.054 (7)	−0.075 (3)	0.513 (3)	0.192*	
O3W	0.0648 (7)	0.4362 (5)	0.5578 (5)	0.181 (3)	
H3B	0.019 (11)	0.389 (2)	0.523 (6)	0.272*	
H3A	0.005 (7)	0.483 (4)	0.5640 (14)	0.272*	
S1	0.10110 (11)	0.49166 (8)	0.80514 (8)	0.0510 (3)	
O4	0.2488 (10)	0.4775 (7)	0.7857 (5)	0.064 (2)	0.676 (12)
O5	0.0049 (6)	0.5169 (5)	0.7424 (5)	0.076 (3)	0.676 (12)
O6	0.0864 (8)	0.5626 (4)	0.8905 (4)	0.080 (2)	0.676 (12)
O6'	0.0143 (13)	0.5620 (9)	0.8224 (13)	0.090 (8)	0.324 (12)
O5'	0.125 (2)	0.4631 (10)	0.7031 (8)	0.107 (7)	0.324 (12)
O4'	0.253 (2)	0.4992 (18)	0.8326 (15)	0.094 (8)	0.324 (12)

### Atomic displacement parameters (Å^2^)

**Table:**

	*U*^11^	*U*^22^	*U*^33^	*U*^12^	*U*^13^	*U*^23^
Ag1	0.0790 (2)	0.04833 (19)	0.03198 (15)	0.00432 (15)	0.00483 (14)	0.00901 (13)
Ag2	0.1229 (3)	0.0504 (2)	0.03425 (17)	0.00170 (19)	0.01163 (18)	0.01212 (14)
C1	0.0295 (17)	0.046 (2)	0.0401 (18)	−0.0052 (14)	−0.0040 (15)	0.0122 (16)
C2	0.0265 (16)	0.052 (2)	0.044 (2)	−0.0043 (15)	0.0026 (15)	0.0138 (17)
C3	0.0359 (19)	0.047 (2)	0.0415 (19)	−0.0066 (16)	−0.0045 (16)	0.0081 (17)
C4	0.046 (2)	0.054 (2)	0.046 (2)	−0.0101 (18)	−0.0058 (18)	0.0184 (19)
C5	0.049 (2)	0.068 (3)	0.046 (2)	−0.002 (2)	0.0157 (19)	0.020 (2)
C6	0.050 (2)	0.050 (2)	0.046 (2)	0.0058 (18)	0.0076 (18)	0.0086 (18)
C7	0.049 (2)	0.053 (3)	0.086 (3)	−0.004 (2)	−0.003 (2)	−0.004 (2)
C8	0.058 (2)	0.045 (2)	0.046 (2)	−0.0104 (18)	−0.0092 (19)	0.0203 (18)
C9	0.043 (2)	0.089 (4)	0.047 (2)	−0.009 (2)	−0.0020 (19)	0.038 (2)
C10	0.052 (2)	0.092 (4)	0.0321 (19)	0.009 (2)	0.0080 (18)	0.014 (2)
C11	0.056 (2)	0.051 (2)	0.0339 (18)	0.0069 (18)	0.0001 (18)	0.0080 (17)
C12	0.0412 (19)	0.042 (2)	0.0247 (15)	−0.0017 (15)	−0.0018 (14)	0.0107 (14)
C13	0.0413 (19)	0.042 (2)	0.0259 (15)	0.0018 (15)	−0.0058 (14)	0.0090 (14)
C14	0.058 (2)	0.053 (2)	0.0353 (19)	0.0108 (19)	−0.0033 (18)	0.0044 (17)
C15	0.059 (2)	0.052 (2)	0.038 (2)	−0.0156 (19)	−0.0024 (18)	0.0166 (18)
C16	0.045 (2)	0.077 (3)	0.050 (2)	−0.012 (2)	0.0021 (19)	0.030 (2)
C17	0.044 (2)	0.048 (2)	0.0364 (19)	0.0062 (17)	0.0022 (17)	0.0048 (16)
C18	0.037 (2)	0.094 (4)	0.039 (2)	0.002 (2)	−0.0025 (18)	0.005 (2)
C19	0.048 (2)	0.092 (4)	0.037 (2)	0.002 (2)	−0.0084 (19)	−0.002 (2)
C20	0.0400 (19)	0.040 (2)	0.0407 (19)	0.0072 (15)	0.0024 (16)	0.0146 (16)
C21	0.046 (2)	0.107 (4)	0.058 (3)	0.006 (2)	−0.009 (2)	0.040 (3)
C22	0.038 (2)	0.100 (4)	0.062 (3)	0.006 (2)	0.003 (2)	0.043 (3)
C23	0.0415 (19)	0.0361 (19)	0.0362 (17)	−0.0049 (14)	−0.0052 (15)	0.0137 (15)
C24	0.0323 (17)	0.0390 (19)	0.0262 (15)	−0.0001 (14)	−0.0034 (13)	0.0096 (14)
C25	0.047 (2)	0.054 (2)	0.0364 (19)	0.0074 (18)	0.0021 (17)	0.0011 (17)
C26	0.037 (2)	0.101 (4)	0.0346 (19)	0.008 (2)	0.0062 (17)	0.014 (2)
C27	0.046 (2)	0.096 (4)	0.047 (2)	−0.018 (2)	0.004 (2)	0.033 (2)
C28	0.063 (2)	0.048 (2)	0.040 (2)	−0.0165 (19)	−0.0027 (19)	0.0183 (18)
C29	0.0394 (18)	0.0397 (19)	0.0252 (15)	−0.0039 (14)	−0.0030 (14)	0.0098 (14)
C30	0.050 (2)	0.042 (2)	0.0331 (17)	0.0053 (16)	−0.0042 (16)	0.0061 (15)
C31	0.046 (2)	0.078 (3)	0.042 (2)	0.002 (2)	0.0054 (19)	0.005 (2)
C32	0.056 (3)	0.074 (3)	0.038 (2)	0.010 (2)	−0.0082 (19)	0.003 (2)
C33	0.058 (2)	0.036 (2)	0.041 (2)	−0.0033 (17)	0.0071 (18)	0.0063 (16)
C34	0.059 (3)	0.070 (3)	0.049 (2)	−0.018 (2)	−0.009 (2)	0.023 (2)
C35	0.063 (3)	0.046 (2)	0.041 (2)	−0.0007 (18)	0.0079 (19)	0.0134 (18)
C36	0.071 (3)	0.087 (4)	0.075 (3)	0.013 (3)	0.006 (3)	0.020 (3)
N1	0.0487 (18)	0.0468 (18)	0.0375 (16)	−0.0093 (14)	−0.0003 (14)	0.0181 (14)
N2	0.0461 (17)	0.0435 (18)	0.0306 (15)	0.0050 (13)	−0.0009 (13)	0.0019 (13)
N3	0.0539 (19)	0.0479 (19)	0.0296 (15)	0.0069 (14)	−0.0006 (14)	0.0020 (13)
N4	0.074 (2)	0.049 (2)	0.0354 (16)	−0.0094 (17)	−0.0016 (17)	0.0148 (15)
N5	0.059 (2)	0.0443 (18)	0.0344 (16)	0.0077 (14)	0.0006 (15)	0.0135 (14)
N6	0.0365 (15)	0.0330 (15)	0.0354 (15)	0.0027 (12)	−0.0013 (13)	0.0139 (12)
N7	0.071 (2)	0.0445 (19)	0.0368 (17)	0.0072 (16)	0.0039 (16)	0.0073 (14)
N8	0.0454 (17)	0.0362 (16)	0.0320 (15)	0.0060 (13)	0.0005 (13)	0.0035 (12)
O1	0.077 (2)	0.0436 (19)	0.118 (3)	−0.0043 (16)	−0.014 (2)	0.0087 (19)
O2	0.095 (3)	0.074 (3)	0.172 (5)	−0.005 (2)	0.076 (3)	−0.025 (3)
O3	0.081 (2)	0.066 (2)	0.075 (2)	−0.027 (2)	0.0109 (19)	0.0296 (19)
O7	0.0474 (16)	0.064 (2)	0.070 (2)	−0.0004 (15)	0.0075 (15)	0.0127 (17)
O1W	0.139 (4)	0.184 (6)	0.093 (4)	0.007 (4)	0.005 (3)	0.026 (4)
O2W	0.144 (4)	0.152 (5)	0.089 (3)	0.038 (4)	0.023 (3)	0.026 (3)
O3W	0.181 (6)	0.162 (6)	0.179 (7)	−0.033 (5)	0.039 (5)	−0.010 (5)
S1	0.0394 (5)	0.0466 (6)	0.0711 (7)	−0.0050 (4)	−0.0040 (5)	0.0238 (5)
O4	0.036 (4)	0.057 (4)	0.104 (6)	0.000 (3)	0.022 (5)	0.027 (4)
O5	0.064 (4)	0.076 (4)	0.102 (5)	−0.016 (3)	−0.029 (4)	0.056 (4)
O6	0.122 (5)	0.041 (3)	0.067 (4)	−0.019 (3)	0.025 (4)	−0.009 (2)
O6'	0.066 (8)	0.056 (7)	0.17 (2)	0.030 (6)	0.050 (12)	0.053 (10)
O5'	0.165 (17)	0.095 (10)	0.079 (9)	0.003 (10)	0.026 (9)	0.056 (8)
O4'	0.048 (8)	0.092 (15)	0.160 (19)	−0.027 (9)	−0.046 (13)	0.078 (15)

### Geometric parameters (Å, °)

**Table:**

Ag1—N5	2.105 (3)	C21—H21	0.93
Ag1—N3	2.121 (3)	C22—N6	1.364 (5)
Ag2—N4	2.089 (3)	C22—H22	0.93
Ag2—N7	2.091 (3)	C23—N6	1.474 (4)
C1—C2	1.363 (5)	C23—C24	1.506 (4)
C1—C6	1.393 (5)	C23—H23A	0.97
C1—S1	1.770 (3)	C23—H23B	0.97
C2—C3	1.387 (5)	C24—C29	1.387 (5)
C2—H2	0.93	C24—C25	1.392 (5)
C3—C4	1.397 (5)	C25—C26	1.388 (6)
C3—C7	1.500 (6)	C25—H25	0.93
C4—O3	1.354 (5)	C26—C27	1.355 (7)
C4—C5	1.374 (6)	C26—H26	0.93
C5—C6	1.379 (5)	C27—C28	1.374 (6)
C5—H5	0.93	C27—H27	0.93
C6—H6	0.93	C28—C29	1.393 (4)
C7—O2	1.233 (6)	C28—H28	0.93
C7—O1	1.263 (5)	C29—C30	1.505 (5)
C8—C9	1.375 (6)	C30—N8	1.475 (4)
C8—C13	1.380 (5)	C30—H30A	0.97
C8—H8	0.93	C30—H30B	0.97
C9—C10	1.368 (6)	C31—C32	1.332 (5)
C9—H9	0.93	C31—N8	1.372 (5)
C10—C11	1.389 (5)	C31—H31	0.93
C10—H10	0.93	C32—N7	1.361 (5)
C11—C12	1.383 (5)	C32—H32	0.93
C11—H11	0.93	C33—N7	1.323 (5)
C12—C13	1.387 (5)	C33—N8	1.333 (5)
C12—C15	1.513 (4)	C33—H33	0.93
C13—C14	1.506 (5)	C34—N4	1.365 (5)
C14—N2	1.467 (4)	C34—H36	0.93
C14—H14A	0.97	C35—N4	1.317 (5)
C14—H14B	0.97	C35—N1	1.334 (5)
C15—N1	1.466 (5)	C35—H324	0.93
C15—H15A	0.97	C36—O7	1.416 (6)
C15—H15B	0.97	C36—H36A	0.96
C16—N1	1.355 (5)	C36—H36B	0.96
C16—C34	1.361 (5)	C36—H36C	0.96
C16—H35	0.93	O3—H4A	0.86 (4)
C17—N3	1.311 (5)	O7—H7A	0.842 (10)
C17—N2	1.341 (4)	O1W—H1A	0.856 (10)
C17—H17	0.93	O1W—H1B	0.85 (6)
C18—C19	1.343 (5)	O2W—H2A	0.86 (5)
C18—N2	1.363 (5)	O2W—H2B	0.86 (5)
C18—H18	0.93	O3W—H3B	0.85 (7)
C19—N3	1.360 (5)	O3W—H3A	0.85 (6)
C19—H19	0.93	S1—O6'	1.258 (10)
C20—N5	1.326 (5)	S1—O4	1.386 (9)
C20—N6	1.332 (4)	S1—O5	1.407 (5)
C20—H20	0.93	S1—O4'	1.42 (2)
C21—C22	1.330 (6)	S1—O6	1.514 (5)
C21—N5	1.351 (5)	S1—O5'	1.619 (13)
			
N5—Ag1—N3	176.76 (13)	C27—C26—H26	120.0
N4—Ag2—N7	172.60 (13)	C25—C26—H26	120.0
C2—C1—C6	119.5 (3)	C26—C27—C28	120.4 (3)
C2—C1—S1	120.1 (3)	C26—C27—H27	119.8
C6—C1—S1	120.4 (3)	C28—C27—H27	119.8
C1—C2—C3	121.9 (3)	C27—C28—C29	120.7 (4)
C1—C2—H2	119.1	C27—C28—H28	119.7
C3—C2—H2	119.1	C29—C28—H28	119.7
C2—C3—C4	118.2 (4)	C24—C29—C28	119.4 (3)
C2—C3—C7	120.9 (4)	C24—C29—C30	122.4 (3)
C4—C3—C7	120.9 (4)	C28—C29—C30	118.2 (3)
O3—C4—C5	119.2 (4)	N8—C30—C29	111.6 (3)
O3—C4—C3	120.7 (4)	N8—C30—H30A	109.3
C5—C4—C3	120.2 (3)	C29—C30—H30A	109.3
C4—C5—C6	120.8 (4)	N8—C30—H30B	109.3
C4—C5—H5	119.6	C29—C30—H30B	109.3
C6—C5—H5	119.6	H30A—C30—H30B	108.0
C5—C6—C1	119.5 (4)	C32—C31—N8	106.3 (4)
C5—C6—H6	120.2	C32—C31—H31	126.8
C1—C6—H6	120.2	N8—C31—H31	126.8
O2—C7—O1	124.5 (5)	C31—C32—N7	110.2 (4)
O2—C7—C3	118.7 (4)	C31—C32—H32	124.9
O1—C7—C3	116.8 (4)	N7—C32—H32	124.9
C9—C8—C13	121.6 (4)	N7—C33—N8	110.8 (3)
C9—C8—H8	119.2	N7—C33—H33	124.6
C13—C8—H8	119.2	N8—C33—H33	124.6
C10—C9—C8	119.8 (3)	C16—C34—N4	108.6 (4)
C10—C9—H9	120.1	C16—C34—H36	125.7
C8—C9—H9	120.1	N4—C34—H36	125.7
C9—C10—C11	119.6 (4)	N4—C35—N1	111.0 (4)
C9—C10—H10	120.2	N4—C35—H324	124.5
C11—C10—H10	120.2	N1—C35—H324	124.5
C12—C11—C10	120.6 (4)	O7—C36—H36A	109.5
C12—C11—H11	119.7	O7—C36—H36B	109.5
C10—C11—H11	119.7	H36A—C36—H36B	109.5
C11—C12—C13	119.6 (3)	O7—C36—H36C	109.5
C11—C12—C15	118.3 (3)	H36A—C36—H36C	109.5
C13—C12—C15	122.1 (3)	H36B—C36—H36C	109.5
C8—C13—C12	118.8 (3)	C35—N1—C16	107.4 (3)
C8—C13—C14	119.2 (3)	C35—N1—C15	125.4 (4)
C12—C13—C14	122.0 (3)	C16—N1—C15	127.1 (3)
N2—C14—C13	111.9 (3)	C17—N2—C18	106.6 (3)
N2—C14—H14A	109.2	C17—N2—C14	126.4 (3)
C13—C14—H14A	109.2	C18—N2—C14	127.0 (3)
N2—C14—H14B	109.2	C17—N3—C19	106.0 (3)
C13—C14—H14B	109.2	C17—N3—Ag1	127.2 (3)
H14A—C14—H14B	107.9	C19—N3—Ag1	126.6 (3)
N1—C15—C12	112.1 (3)	C35—N4—C34	106.2 (3)
N1—C15—H15A	109.2	C35—N4—Ag2	124.1 (3)
C12—C15—H15A	109.2	C34—N4—Ag2	129.6 (3)
N1—C15—H15B	109.2	C20—N5—C21	104.6 (3)
C12—C15—H15B	109.2	C20—N5—Ag1	130.7 (3)
H15A—C15—H15B	107.9	C21—N5—Ag1	124.5 (3)
N1—C16—C34	106.7 (4)	C20—N6—C22	107.2 (3)
N1—C16—H35	126.7	C20—N6—C23	126.2 (3)
C34—C16—H35	126.7	C22—N6—C23	126.6 (3)
N3—C17—N2	111.2 (3)	C33—N7—C32	105.6 (3)
N3—C17—H17	124.4	C33—N7—Ag2	126.2 (3)
N2—C17—H17	124.4	C32—N7—Ag2	128.2 (3)
C19—C18—N2	106.7 (4)	C33—N8—C31	107.1 (3)
C19—C18—H18	126.6	C33—N8—C30	125.6 (3)
N2—C18—H18	126.6	C31—N8—C30	127.3 (3)
C18—C19—N3	109.5 (4)	C4—O3—H4A	108 (4)
C18—C19—H19	125.3	C36—O7—H7A	108 (3)
N3—C19—H19	125.3	H1A—O1W—H1B	104 (6)
N5—C20—N6	111.1 (3)	H2A—O2W—H2B	104 (5)
N5—C20—H20	124.4	H3B—O3W—H3A	105 (7)
N6—C20—H20	124.4	O6'—S1—O4	138.1 (6)
C22—C21—N5	111.3 (4)	O6'—S1—O5	57.8 (9)
C22—C21—H21	124.3	O4—S1—O5	117.4 (5)
N5—C21—H21	124.3	O6'—S1—O4'	123.3 (13)
C21—C22—N6	105.7 (4)	O5—S1—O4'	140.5 (9)
C21—C22—H22	127.1	O6'—S1—O6	52.4 (9)
N6—C22—H22	127.1	O4—S1—O6	111.7 (4)
N6—C23—C24	111.8 (3)	O5—S1—O6	110.1 (5)
N6—C23—H23A	109.2	O4'—S1—O6	82.2 (10)
C24—C23—H23A	109.2	O6'—S1—O5'	110.1 (10)
N6—C23—H23B	109.2	O4—S1—O5'	66.9 (7)
C24—C23—H23B	109.2	O5—S1—O5'	55.4 (6)
H23A—C23—H23B	107.9	O4'—S1—O5'	97.3 (10)
C29—C24—C25	118.9 (3)	O6—S1—O5'	154.3 (5)
C29—C24—C23	122.8 (3)	O6'—S1—C1	115.0 (5)
C25—C24—C23	118.3 (3)	O4—S1—C1	106.3 (4)
C26—C25—C24	120.6 (4)	O5—S1—C1	106.6 (2)
C26—C25—H25	119.7	O4'—S1—C1	106.4 (9)
C24—C25—H25	119.7	O6—S1—C1	103.6 (2)
C27—C26—C25	120.0 (4)	O5'—S1—C1	101.2 (5)
			
C6—C1—C2—C3	0.3 (5)	N4—C35—N1—C15	−179.0 (3)
S1—C1—C2—C3	−178.2 (3)	C34—C16—N1—C35	−0.3 (5)
C1—C2—C3—C4	1.2 (5)	C34—C16—N1—C15	178.8 (4)
C1—C2—C3—C7	−179.5 (4)	C12—C15—N1—C35	−133.8 (4)
C2—C3—C4—O3	178.4 (3)	C12—C15—N1—C16	47.3 (5)
C7—C3—C4—O3	−0.9 (6)	N3—C17—N2—C18	1.3 (5)
C2—C3—C4—C5	−1.9 (6)	N3—C17—N2—C14	−177.9 (3)
C7—C3—C4—C5	178.8 (4)	C19—C18—N2—C17	−1.4 (5)
O3—C4—C5—C6	−179.1 (4)	C19—C18—N2—C14	177.8 (4)
C3—C4—C5—C6	1.2 (6)	C13—C14—N2—C17	151.7 (3)
C4—C5—C6—C1	0.3 (6)	C13—C14—N2—C18	−27.4 (6)
C2—C1—C6—C5	−1.1 (6)	N2—C17—N3—C19	−0.7 (5)
S1—C1—C6—C5	177.4 (3)	N2—C17—N3—Ag1	174.1 (2)
C2—C3—C7—O2	−1.7 (7)	C18—C19—N3—C17	−0.2 (5)
C4—C3—C7—O2	177.6 (5)	C18—C19—N3—Ag1	−175.0 (3)
C2—C3—C7—O1	−179.3 (4)	N1—C35—N4—C34	0.2 (4)
C4—C3—C7—O1	−0.1 (6)	N1—C35—N4—Ag2	178.2 (2)
C13—C8—C9—C10	−0.3 (6)	C16—C34—N4—C35	−0.4 (5)
C8—C9—C10—C11	0.1 (6)	C16—C34—N4—Ag2	−178.3 (3)
C9—C10—C11—C12	0.0 (6)	N6—C20—N5—C21	−0.5 (4)
C10—C11—C12—C13	0.2 (5)	N6—C20—N5—Ag1	176.2 (2)
C10—C11—C12—C15	179.4 (3)	C22—C21—N5—C20	0.0 (6)
C9—C8—C13—C12	0.5 (5)	C22—C21—N5—Ag1	−176.9 (3)
C9—C8—C13—C14	−178.5 (3)	N5—C20—N6—C22	0.7 (4)
C11—C12—C13—C8	−0.4 (5)	N5—C20—N6—C23	−179.3 (3)
C15—C12—C13—C8	−179.6 (3)	C21—C22—N6—C20	−0.7 (5)
C11—C12—C13—C14	178.6 (3)	C21—C22—N6—C23	179.4 (4)
C15—C12—C13—C14	−0.6 (5)	C24—C23—N6—C20	140.9 (3)
C8—C13—C14—N2	98.4 (4)	C24—C23—N6—C22	−39.1 (5)
C12—C13—C14—N2	−80.5 (4)	N8—C33—N7—C32	1.0 (4)
C11—C12—C15—N1	−94.9 (4)	N8—C33—N7—Ag2	179.6 (2)
C13—C12—C15—N1	84.3 (4)	C31—C32—N7—C33	−0.8 (5)
N2—C18—C19—N3	1.0 (6)	C31—C32—N7—Ag2	−179.3 (3)
N5—C21—C22—N6	0.4 (6)	N7—C33—N8—C31	−0.9 (4)
N6—C23—C24—C29	−83.6 (4)	N7—C33—N8—C30	179.8 (3)
N6—C23—C24—C25	97.4 (4)	C32—C31—N8—C33	0.3 (5)
C29—C24—C25—C26	0.0 (5)	C32—C31—N8—C30	179.7 (4)
C23—C24—C25—C26	179.0 (3)	C29—C30—N8—C33	−133.9 (3)
C24—C25—C26—C27	0.3 (6)	C29—C30—N8—C31	46.9 (5)
C25—C26—C27—C28	−1.0 (6)	C2—C1—S1—O6'	154.7 (11)
C26—C27—C28—C29	1.4 (6)	C6—C1—S1—O6'	−23.8 (11)
C25—C24—C29—C28	0.4 (5)	C2—C1—S1—O4	−33.0 (5)
C23—C24—C29—C28	−178.5 (3)	C6—C1—S1—O4	148.5 (5)
C25—C24—C29—C30	179.9 (3)	C2—C1—S1—O5	93.0 (5)
C23—C24—C29—C30	0.9 (5)	C6—C1—S1—O5	−85.5 (5)
C27—C28—C29—C24	−1.1 (5)	C2—C1—S1—O4'	−65.1 (11)
C27—C28—C29—C30	179.4 (3)	C6—C1—S1—O4'	116.4 (11)
C24—C29—C30—N8	84.9 (4)	C2—C1—S1—O6	−150.8 (4)
C28—C29—C30—N8	−95.6 (4)	C6—C1—S1—O6	30.7 (5)
N8—C31—C32—N7	0.3 (5)	C2—C1—S1—O5'	36.1 (7)
N1—C16—C34—N4	0.4 (5)	C6—C1—S1—O5'	−142.4 (7)
N4—C35—N1—C16	0.1 (4)		

### Hydrogen-bond geometry (Å, °)

**Table:**

*D*—H···*A*	*D*—H	H···*A*	*D*···*A*	*D*—H···*A*
O1W—H1B···O3W	0.85 (6)	2.35 (5)	2.892 (9)	122 (4)
O2W—H2A···O1	0.86 (5)	2.52 (6)	3.063 (7)	122 (6)
O3—H4A···O1	0.86 (4)	1.71 (3)	2.486 (6)	149 (5)
O7—H7A···O6^i^	0.84 (1)	1.88 (2)	2.697 (6)	164 (4)
O2W—H2B···O1W^ii^	0.86 (5)	2.24 (5)	2.790 (9)	122 (4)
O3W—H3A···O3W^iii^	0.85 (6)	2.52 (4)	3.053 (10)	122 (2)

Symmetry codes: (i) *x*, *y*, *z*−1; (ii) −*x*, −*y*, −*z*+1; (iii) −*x*, −*y*+1, −*z*+1.

## References

[bb1] Cote, A. P. & Shimizu, G. K. H. (2003). *Coord. Chem. Rev.* **245**, 49–64.

[bb2] Cote, A. P. & Shimizu, G. K. H. (2004). *Inorg. Chem.* **43**, 6663–6673. 10.1021/ic0491229 15476366

[bb3] Higashi, T. (1995). *ABSCOR* Rigaku Corporation, Tokyo, Japan.

[bb4] Li, F.-F., Ma, J.-F., Song, S.-Y., Yang, J., Jia, H.-Q. & Hu, N.-H. (2006). *Cryst. Growth Des.* **6**, 209–215.

[bb5] Liu, H.-Y., Wu, H., Ma, J.-F., Song, S.-Y., Yang, J., Liu, Y.-Y. & Su, Z.-M. (2007). *Inorg. Chem.* **46**, 7299–7311. 10.1021/ic070147s 17685508

[bb6] Ma, J.-F., Yang, J., Li, S.-L., Song, S.-Y., Zhang, H.-J., Wang, H.-S. & Yang, K.-Y. (2005). *Cryst. Growth Des.* **5**, 807–812.

[bb7] Rigaku (1998). *PROCESS-AUTO* Rigaku Corporation, Tokyo, Japan.

[bb8] Sheldrick, G. M. (2008). *Acta Cryst.* A**64**, 112–122. 10.1107/S0108767307043930 18156677

